# Artificial intelligence in arrhythmia risk prediction: connecting undetectable vulnerabilities and proactive care

**DOI:** 10.1097/MS9.0000000000004562

**Published:** 2025-12-12

**Authors:** Asra Amjad, Maryam Altaf Hussain, Umair Ali, Iman Osman Abufatima

**Affiliations:** a Department of Medicine, Islamic International Medical College Rawalpindi Pakistan (Riphah International University); b Department of Medicine, Karachi Metropolitan University; c Department of Pharmacy, University of Swabi, Pakistan; d Department of Medicine, University of Medical Sciences and Technology, Khartoum, Sudan

*To the Editor,*

Arrhythmias provide a clinical challenge not just because they appear suddenly but also because of their silent, subclinical stages, which conceal risk from conventional diagnostic methods. With artificial intelligence (AI) bridging the gap between early detection and invisibility, cardiology now has a new preventive dimension. Even seemingly normal electrocardiograms (ECGs) can reveal subclinical electrical and structural problems, such as latent arrhythmias or prolonged QT, thanks to AI, especially deep neural networks^[1,2]^. AI’s function as a disruptive supplement to traditional cardiology is justified by this fundamental evidence, which shows how it reveals information that professionals cannot notice on surface ECGs.

Atrial fibrillation (AF) screening at the population level is made easier by wearable technology that uses photoplethysmography and machine learning^[1,2]^. The transition from static ECGs to ongoing, population-level monitoring highlights AI’s scalability, guaranteeing that detection is not limited to hospital settings but rather permeates daily life. Even in sinus rhythm, AI may identify minor indicators of susceptibility such P-wave microfluctuations. When integrated into electrophysiology, it allows for the early assessment of the risk of sudden cardiac death, the diagnosis of arrhythmias, and the optimization of medication^[1]^. This demonstrates how micro-level data can influence macro-level results in patient safety and prevention, establishing the direct therapeutic relevance of AI.

True anticipatory arrhythmia risk classification is promised by this paradigm shift toward mobile, personalized cardiology, but such assurances require proof. New models make an effort to verify these possibilities and improve their practical implementation. The potential of ECG micro-pattern recognition for preclinical arrhythmia risk stratification is highlighted by AI-enabled models that use the electrocardiogram waveform to extract predictive information that is additive to conventional clinical risk factors, allowing for the earlier and more effective identification of people at risk of AF^[2,3]^. Naturally, this prepares the ground for examining whether these models may surpass current clinical scores, a question that has been the subject of recent extensive research.

Using 12-lead sinus-rhythm ECGs, Yuan *et al* created a deep-learning model that accurately identified AF within 31 days area under the receiver operating characteristic curve (AUROC) 0.86 in ventricular arrhythmia, 0.93 in external cohorts) and consistently maintained accuracy (AUROC 0.80–0.85) within a year of follow-up^[3]^. Strong measures like these show not only technical proficiency but also the forecasts’ longevity over clinically significant time periods. Crucially, this AI-ECG model reduced the number required to screen from approximately 11 to approximately 2–3, outperforming CHA_2_DS_2_-VASc and other common clinical techniques^[3,4]^. The model’s generalizability is supported by its consistent performance across a range of demographic and comorbidity profiles. According to these findings, AI can supplement and even outperform current risk classification techniques by bridging the gap between statistical power and clinical practicality.

With the possibility to include data from wearable formats (such as Holter or smartwatch ECG) and echocardiography into ensemble frameworks for more accurate preemptive arrhythmia risk classification, such AI models may aid in patient stratification for focused intense monitoring programs^[4]^. The idea that arrhythmia prevention involves dynamic integration across technologies rather than a single test is reinforced by the convergence of multimodal data.

By facilitating early detection using wearables and AI-ECG, improving anticoagulation choices, streamlining ablation techniques, and facilitating continuous monitoring through multimodal data integration, AI is driving a preventive paradigm shift in the treatment of AF^[4]^. In addition to these technological advances, medicine has to deal with the inevitable conflict between creativity and moral obligation. The ethical and therapeutic issues of overdiagnosis, overtreatment, equity, and the rationale for preventative measures in asymptomatic patients are brought up by AI-based early prediction of subclinical AF^[5]^. These issues show that predictive capacity is only useful when it is incorporated into ethical clinical frameworks.

By targeting high-risk people for ECG monitoring using a machine learning risk algorithm, the PULsE-AI experiment provides a more effective technique to identify asymptomatic AF and facilitate preventive intervention^[6]^. This illustrates how AI-powered targeted screening can convert prediction into practical treatment rather than just theoretical likelihood. Seasonal variations, air pollution, noise, altitude, and work-related stressors are among the environmental exposures that are increasingly understood to trigger AF through autonomic, inflammatory, and oxidative pathways. By incorporating such environmental and temporal data into AI prediction models, context-aware risk stratification and preventive management may be enhanced^[7]^.

The future of AI cardiology is reflected in the inclusion of such external variables, which contextualize cardiac disease within the patient’s lived environment in addition to forecasting it.

In conclusion, the paradigm change from reaction to prevention is exemplified by AI’s capacity to contextualize arrhythmia risk and identify concealed vulnerabilities. The field’s current issue is to make sure that these tools transform how we identify and manage cardiovascular risk from experimental promise to practically applicable, morally sound solutions.

This manuscript complies with TITAN Guidelines, 2025, declaring no use of AI^[8]^.

## Data Availability

All data supporting the findings of this study are available within the article. The study is based on previously published literature; no new data were generated or analyzed.
